# Effect of Relative Humidity on the Electrospinning Performance of Regenerated Silk Solution

**DOI:** 10.3390/polym13152479

**Published:** 2021-07-28

**Authors:** Bo Kyung Park, In Chul Um

**Affiliations:** Department of Biofibers and Biomaterials Science, Kyungpook National University, Daegu 41566, Korea; bkpark645@unm.edu

**Keywords:** silk, electrospinning, relative humidity, morphology, maximum electrospinning rate, molecular conformation

## Abstract

Recently, the electrospun silk web has been intensively studied in terms of its biomedical applications, including tissue engineering scaffolds, due to its good biocompatibility, cytocompatibility, and biodegradability. In this study, the effect of relative humidity (RH) conditions on the morphology of electrospun silk fiber and the electrospinning production rate of silk solution was examined. In addition, the effect of RH on the molecular conformation of electrospun silk web was examined using Fourier transform infrared (FTIR) spectroscopy. As RH was increased, the maximum electrospinning rate of silk solution and fiber diameter of the resultant electrospun silk web were decreased. When RH was increased to 60%, some beads were observed, which showed that the electrospinnability of silk formic acid solution deteriorated with an increase in RH. The FTIR results showed that electrospun silk web was partially β-sheet crystallized and RH did not affect the molecular conformation of silk.

## 1. Introduction

Silk is a naturally occurring polymeric material that has long been used as the best textile material. Recently, the unique properties of silk, including compatibility with blood [1,2], low inflammatory reaction in the body [3], and good cytocompatibility [4], have attracted the attention of researchers in the biomedical field with interests in such applications as artificial eardrums [5,6], tissue engineering scaffolds [7,8,9,10], and wound dressings [11,12].

Electrospinning is a simple method to produce non-woven webs with nanosized fiber diameters [13,14,15,16,17]. Thus, owing to the good biocompatibility of silk and easy nanoweb production by electrospinning, the electrospun silk web has been used in tissue engineering scaffolds, and the number of related studies has recently increased significantly.

The morphological properties of the electrospun web, including fiber diameter, pore size, and fiber alignment, play an essential role in cell adhesion and proliferation for tissue engineering scaffold applications [18,19]. Thus, the parameters influencing the electrospinning performance and the resultant fiber morphology of silk have been extensively studied. The effects of parameters such as silk concentration [20,21,22,23,24,25,26], the PH of the dope solution [21], applied voltage [20,22,23,25], tip to collector distance [20], spinning temperature [23], the feed rate of the dope solution [22,24], the sericin content in silk [24,27,28], molecular weight (MW) [25,29] and the storage time of the silk solution [29], degumming method [30], and the silkworm variety [31] have been previously studied.

It has been reported that relative humidity (RH) is one of the processing parameters affecting the morphology of electrospun fiber [32,33,34,35,36,37,38,39,40,41,42,43,44,45]. The effects of RH on the morphology of electrospun fiber are diverse. Some studies reported that pores in electrospun fiber surfaces are generated and the pore size increased with an increase in RH [32,33,37,44,45]. The effects of RH on the tendencies in the diameter of electrospun fiber were quite different depending on the polymer and solvent system [32,33,34,35,36,37,38,39,40,41,42,43,44,45]. In addition, the productivity and morphology of electrospun fiber were changed by different RH values. For instance, it has been reported that the productivity of electrospun fiber was increased [34,44], and that bead formation was decreased [35] or increased [33,39], with an increase in RH. These studies show that the effect of RH on the electrospinning performance and electrospun-morphology of the resultant fiber is diverse and depends on the types of polymer and solvent used for electrospinning. However, the effect of RH on the electrospinning performance and electrospun morphology of silk formic acid solution has not been examined.

The importance of the maximum electrospinning rate for mass production has long been reported as a means of finding ways to lower the production cost of silk nanoweb [24,25,26]. That is, a relatively low value of maximum electrospinning rate of the polymer has been an obstacle to the mass production and commercialization of the electrospun web. Silk is under the same situation considering that high MW silk resulted in an electrospinning rate of 0.2–0.3 mL/h [27,29,46]. Recently, we learned that the maximum electrospinning rate could be remarkably increased (5–13 fold) by controlling residual sericin content, electric field, silk concentration, and MW of silk [24,25,26].

In this study, the effect of RH on the electrospinning performance of silk formic acid solution was examined by measuring (1) the fiber morphology, including the fiber diameter, (2) the maximum electrospinning rate, and (3) the viscosity of the dope solution. In addition, the effect of RH on the molecular conformation and crystallinity of the resultant electrospun silk webs was examined.

## 2. Experimental

### 2.1. Preparation of Regenerated Silk

The method of preparing regenerated silk has been reported elsewhere [28,47,48,49,50]. Briefly, Bombyx mori silk cocoons were degummed for 1 h using a 0.024% (*w*/*v*) sodium oleate and 0.016% (*w*/*v*) sodium carbonate aqueous solution to obtain silk with a degumming ratio of 19.5% (8.2% residual sericin content). The liquor ratio was 1:25. The degummed silk was dissolved in a ternary solvent, CaCl_2_/H_2_O/EtOH (1/8/2 molar ratio), for 30 min at 85 °C. The liquor ratio was 1:20. The regenerated silk aqueous solution was prepared by dialysis of the dissolved solution (in the ternary solvent) in a cellulose tube (M_W_ cutoff = 12,000–14,000 Da) against purified water for 5 days at 25 °C. Purified water was obtained using a water purification system (RO50, Hanascience, Hanam, South Korea) with a reverse osmosis membrane. The aqueous silk solution was filtered and dried to obtain the regenerated silk powder.

### 2.2. Electrospinning of Regenerated Silk Solution

The regenerated silk powder was dissolved in formic acid (98%) and filtered twice through non-woven fabrics to prepare the dope solution for electrospinning. Silk formic acid solution of 14–19% (*w*/*w*) was loaded into a plastic syringe with a 22 gage stainless steel needle (inner diameter = 0.337 mm) at the tip. Electrospinning was performed using an electrospinning system (NNC-ESP200, NanoNC, Seoul, Korea) at constant temperature (25 ± 1 °C) and RH (20% ± 2%, 40% ± 2%, and 60% ± 2%). The applied voltage and the tip to collector distance were 20 kV and 19 cm, respectively.

### 2.3. Measurement and Characterization

The maximum electrospinning rate of the dope solution was determined as reported in the previous study [24,25,26]. Briefly, the feed rate of the dope solution was controlled by using a syringe pump (KDS100, KDScientific, Holliston, MA, USA) and was increased until the electrified jet was kept stable (e.g., Taylor cone stability and continuous spinning without dripping were reached). The quality of the electrospun fibers with the maximum spinning rate was confirmed by scanning electron microscopy (SEM, S-570, Hitachi, Tokyo, Japan).

Solutions of regenerated silk with different silk concentrations were used for the rheological measurements. Shear viscosity was measured using a rheometer (Hakke MARS III, Thermo Fisher Scientific, Waltham, MA, USA) with parallel plate geometry (35 mm diameter of the plate) at a shear rate of 10 s^−1^ and 25 °C.

To examine the electrospinning performance and fiber morphology of the resultant silk web, the electrospun silk web was gold-coated for field emission-scanning electron microscopy (FE-SEM; S-570, Hitachi, Tokyo, Japan), and FE-SEM observation was performed on it [51,52,53]. The mean fiber diameter of silk webs was determined by counting 30 fibers from the SEM images using an image analysis program (DIMIS-PRO 2.0, Siwon Optical Technology, Anyang, Korea).

A Fourier transform infrared (FTIR; Nicolet 380, Thermo Fisher Scientific, Waltham, MA, USA) spectrometer in the attenuated total reflection mode was used to examine the molecular conformation and the crystallinity index of the electrospun silk web. The crystallinity index was calculated from the FTIR spectrum at the intensity ratio of 1260 and 1235 cm^−1^ using the following equation [50,54]. The FTIR measurement was performed seven times and the mean and standard deviation of crystallinity index were calculated using the crystallinity indexes obtained from these seven FTIR measurements.

$$\mathrm{Crystallinity}\;\mathrm{index}\;(\%)\;=\;\frac{A_{1260{\mathrm{cm}}^{-1}}}{A_{1235{\mathrm{cm}}^{-1}}}\;\times \;100$$

where *A*_1235cm^−1^_: absorbance at 1235 cm^−1^ (attributed to random coil conformation related to the amorphous region)

*A*_1260cm^−1^_: absorbance at 1260 cm^−1^ (due to β-sheet crystallite related to the crystalline region).

## 3. Results and Discussion

### 3.1. Effects of Relative Humidity on the Maximum Electrospinning Rate and the Electrospun Morphology of Silk

**Figure 1:**
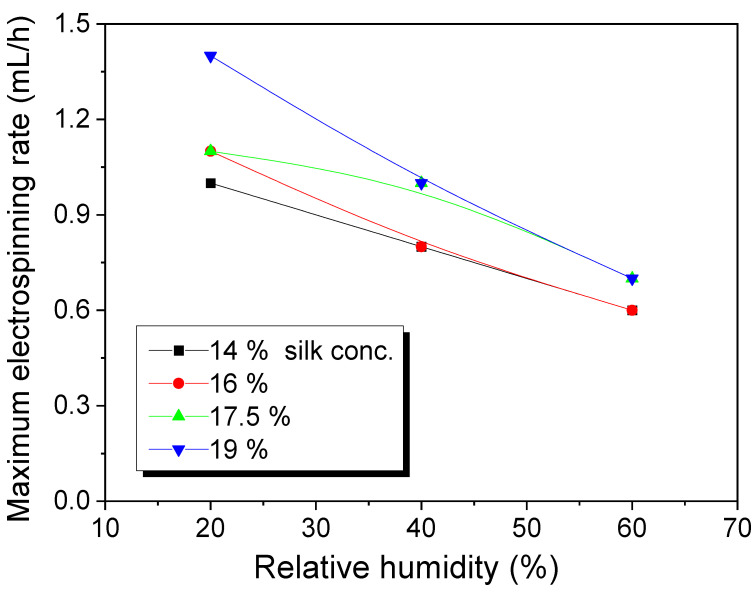
Effect of relative humidity on the maximum electrospinning rate of regenerated silk formic acid solutions with different silk concentrations.

**Figure 2:**
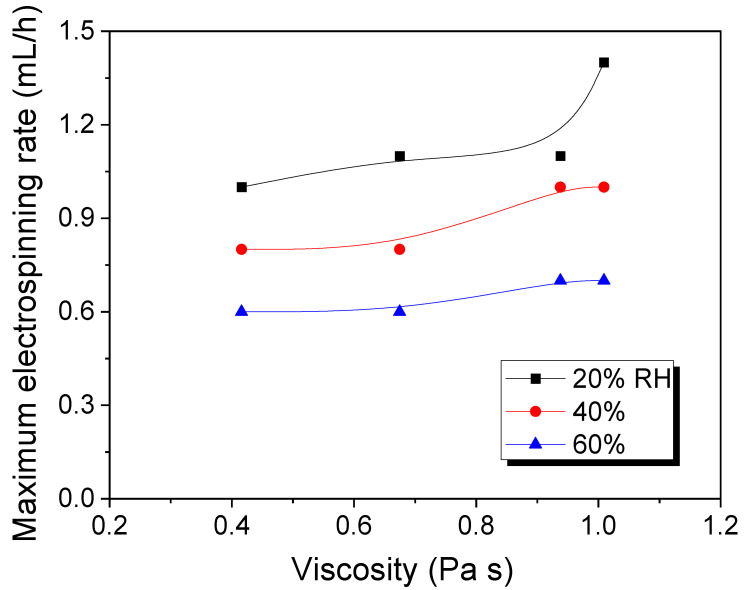
Relationship between relative humidity, viscosity, and maximum electrospinning rate of regenerated silk formic acid solution.

As mentioned in the introduction section, the low electrospinning rate of silk solution (i.e., the low production rate of silk web) has been one of the obstacles to the mass production and commercialization of the electrospun silk web. It is reported that the electrospinning rate can be increased by controlling the degumming ratio at 19.5% (i.e., 8.2% residual sericin content in silk), which is the optimum condition [24]. Thus, in this study, we used regenerated silk with a 19.5% degumming ratio for electrospinning. Figure 1 shows the maximum electrospinning rate of the silk sample at different RH conditions. Regardless of the silk concentration, the maximum electrospinning rate of all silk solutions is decreased with an increase in RH. When the maximum electrospinning rate is plotted against the viscosity of the dope solution, the effect of RH on the electrospinning rate was more clearly observed (Figure 2).

Table 1 shows the morphology of electrospun silk fibers with various silk concentrations produced under different RH values at the maximum electrospinning rate presented in Figure 1 and Figure 2. In the case of 20% and 40% RH conditions, a good fiber formation is observed in all samples regardless of silk concentration (14–19%). However, as the RH is increased to 60%, beads and a little beaded fiber appeared in all silk concentrations, and uneven fiber formation is also observed (particularly at 19% silk concentration). In addition, the bead formation is more noticeable at lower silk concentrations (14–16%).

The results in Table 1 show that the “beads-on-a-string” morphology is more observed with increasing RH. That is, the electrospinnability deteriorates by increasing the RH and decreasing the viscosity. This deterioration of fiber is due to the instability of the Taylor cone and the relaxation time of viscoelastic liquid [55]. The instability on the liquid jet plays an important role in the fiber formation, which directly affects the electrospun fiber morphology. The occurrence of the electrostatic field and surface tension of the polymer solution causes the whipping instability and Rayleigh instability, respectively [40,44]. These instabilities, which are governed by solution properties and processing parameters, such as conductivity, viscosity, surface tension, and the applied electric field, cause bending and stretching of the liquid jet, which is essential for the decrease in fiber diameter. Thus, as RH increases, the jet stability of the silk dope solution deteriorates, resulting in the decrease in Taylor cone stability and increase in bead formation.

It is interesting to note that beads are formed at high RH (60%) and the number of beads formed is increased with decreasing of the silk concentration at 60% RH. This trend follows the previous results [33,39]. When RH increases, the conductivity of the medium is increased by (1) the polarization of air due to a high applied electric field and (2) the formation of conductive “paths” due to water dipoles across the working distance [33]. When the conductivity of the polymer solution is increased, the polymer solution becomes more stretched during electrospinning. However, the conductivity is too high (i.e., when it passes the critical point), which results in the broken spinning solution, resulting in bead formation. Thus, bead formation at 60% RH might be due to an increase in conductivity of the medium water in the air.

In addition, the changes in solvent evaporation rate might lead to the “beads-on-a-string” morphology as well. That is, during the electrospinning process, the viscoelastic properties of the solution are continuously changed due to the ratio changes between the elasticity and plasticity of the polymer solution. The role of elasticity of polymer solution on the electrospinning helps to initiate the jet and prevents it from breaking. The decreased elastic force being prevents the contraction of the jet, which helps jet initiation to occur without breakage [56]. On the other hand, the plasticity allows the polymer chain to keep its conformation and let the jet to continue to be stretched while it travels to the collector. As the elastic forces overcome the plastic forces, fiber begins to form, and for the smooth and no-bead fiber formation, low elasticity and high plasticity are desirable for good fiber formation. When the RH increases, the evaporation rate of the solvent is lowered. That is, the liquid in the jet takes more time to evaporate in the air and this delays the solidification process of fiber. Consequently, as RH increases, the elastic forces slowly overcome the plastic forces, resulting in the formation of the gradual bead.

The increase in bead formation with the decrease in silk concentration was due to a decrease in the viscosity of the dope silk solution. That is, as silk concentration is decreased, the viscosity of the silk solution decreases. When the solution viscosity of the polymer is insufficient, bead formation is more favored as the continuous spinning polymer solution can be more easily broken during the electrospinning process because of the lack of cohesiveness between polymer molecules, as reported in several previous studies [27,29,30].

The decrease in the maximum electrospinning rate of silk by increasing RH could be explained according to a similar reason. That is, as RH increases, the Taylor cone stability is decreased by increasing the conductivity of the medium due to water in the air [40,44]. Thus, as RH increases, the maximum feed rate that maintains good Taylor cone stability will decrease.

Figure 1 shows a slight increase in the maximum electrospinning rate of silk caused by the increasing of the silk concentration. As shown in Figure 2, regardless of RH, all silk solutions present an increase in maximum electrospinning rate with increasing viscosity. This showed that the solution viscosity raises the maximum electrospinning rate of silk. This result is consistent with those of previous reports [24,25,26]. In conclusion, it can be said that the maximum electrospinning rate of silk is strongly affected by RH and dope solution viscosity.

**Figure 3:**
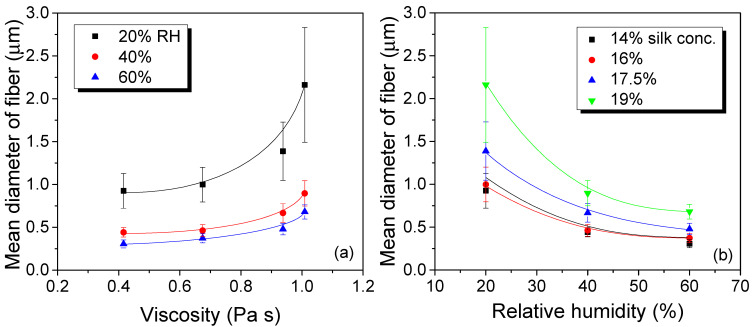
Effect of viscosity (**a**) and relative humidity (**b**) on the mean diameter of electrospun regenerated silk fiber (*n* = 30). The error bars represent the standard deviation.

Figure 3 shows the effects of solution viscosity and RH on the mean diameter of electrospun silk fiber. As shown in the figures, as the solution viscosity increases and the RH decreases, the mean diameter of the electrospun silk fiber increases. The positive relationship between solution viscosity and the mean diameter of the fiber is not surprising, considering this relationship has been reported in several previous studies [26,27,28,29,30,31].

It is interesting to note that the mean diameter of electrospun silk fiber is decreased by the increasing of the RH. Regarding the relationship between the RH and the mean diameter of the electrospun fiber, some studies have been previously performed [32,33,34,35,36,37,38,39,40,41,42,43,44,45]. It has thus been reported that the diameter of electrospun fiber could be increased or decreased by RH depending on the polymer and solvent system. In the electrospinning process, the polymer solution is extruded, forming a droplet that is stretched into a Taylor cone by the voltage applied. If the molecular cohesion of the polymer is sufficiently high, a charged liquid jet is formed and the jet is then elongated by a whipping process, stretching the liquid jet to nano size before the collector. The solvent must evaporate for the polymer to solidify, forming a fiber [35]. The decrease in diameter due to increasing RH is observed in the previous reports on the electrospinning of polyethylene oxide aqueous solution [39] and poly(vinyl pyrrolidone) ethanol solution [36]. In these cases, the evaporation of the solvent is retarded by water present in the air, considering which, the jet can be more elongated, resulting in thinner fiber. The opposite cases are also reported, in which the diameter of electrospun cellulose acetate (CA) and polystyrene (PS) fiber diameter is increased by the increasing of the RH [32,34,37,45]. In the case of CA, it is dissolved in a mixture of acetone and N,N-dimethylacetamide. However, the CA in the mixture solvents rapidly becomes precipitated by water and it impedes jet elongation. Thus, they speculated that CA solution becomes solidified more rapidly by water that is present in the air, resulting in increased fiber diameter [36]. In addition, PS is dissolved in three different solvents: N,N-dimethylformamide (DMF), tetrahydrofuran (THF), and mixtures of THF/DMF. Regardless of the polymer and solvent system, the diameter of PS fiber is increased with an increase in RH. With the increase in RH, more water is penetrated in the polymer solution jet, leading to the increase in viscoelastic forces of the polymer solution jet [37]. Accordingly, the jet elongation is suppressed due to Coulombic forces and whipping instability, leading to the increase in fiber diameter.

In the case of silk, there have been a few studies about the effect of RH on the electrospinning performance. When a silk fibroin and water system are used for electrospinning, the electrospun fiber shows higher inhomogeneity, with an increase in RH from 25% to 30% [57]. When silk fibroin is blended with polyvinyl alcohol (PVA), it shows smoother surfaces and larger diameters with the decreasing of the RH [58]. This is due to the combination of solidification velocity and the bead-on-a-string formation. PVA and silk have different viscoelastic properties and degrees of water absorbability. This may lead to a larger fiber diameter of silk fibroin nanofiber and a smaller fiber diameter of PVA nanofiber when the RH is increased. However, as mentioned earlier, the effects of RH on the electrospinning performance are different depending on the polymer-solvent system. Here, the silk and formic acid systems were used, and it seems that the decrease in diameter caused by the increasing of the RH might be due to discharge, lower evaporation rate, and slower solidification, that are all caused by increasing RH, i.e., the humidity in the silk formic acid system that makes the polymer jet is partially discharged and this is due to the newly created ions in the air by water vapor molecules [59]. Thus, the density of charge on the electrospinning jet is decreased by the increasing of the RH and it constrained the movement of the jet because whipping instability results from the electrostatic repulsion [60]. Thus, the lower number of charges on the electrospinning jet prolongs the jet elongation and lessens the whipping region. In addition, as RH increases, the evaporation of formic acid occurs slowly and fiber solidification is retarded, which also increases the time of jet elongation. This results in a thinner diameter of the fiber and the rousing of “beads-on-string” morphologies that could be observed at high RH. Finally, the mean fiber diameter of electrospun silk decreases with an increase in RH, as shown in Figure 3b.

Conclusively, it can be said that the mean diameter of the electrospun silk web can be diverse when controlling RH. Park and Um reported that the mechanical properties (i.e., strength and elongation) of the silk web are increased by the decreasing of porosity as a result of a decrease in the mean diameter of the electrospun silk [26]. Thus, it is expected that the mechanical properties of the electrospun silk web can also be manipulated by tuning the RH.

**Figure 4:**
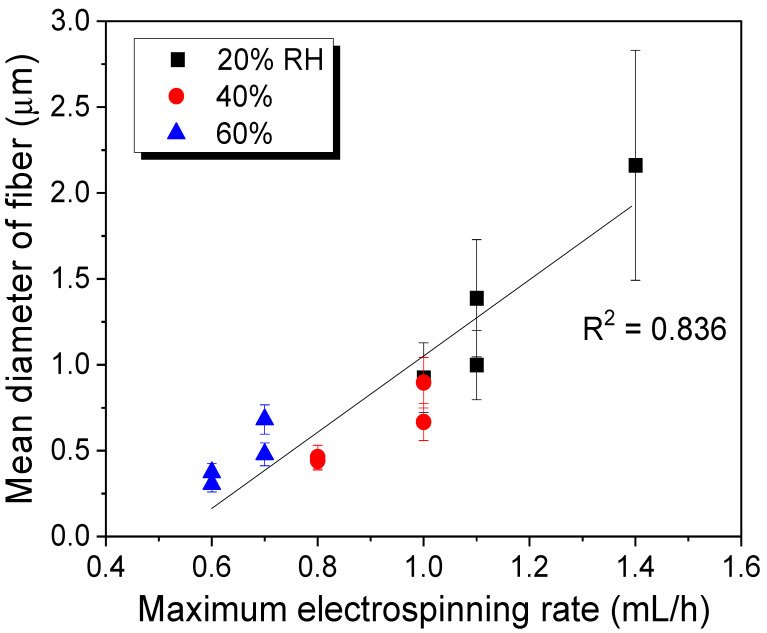
Relationship between the maximum electrospinning rate of silk solution and the fiber diameter of electrospun silk web (*n* = 30). The error bars represent the standard deviation.

As shown in Figure 4, the mean diameter of the fiber of electrospun silk showed a relatively good linear relationship with the maximum electrospinning rate (R^2^ = 0.836). Considering that both the mean diameters and the maximum electrospinning rate have a positive relationship with the dope solution viscosity [26,27,28,29,30,31], their positive linear relationship can easily be rationalized.

### 3.2. Effects of Relative Humidity on the Molecular Conformation of Electrospun Silk Web

**Figure 5:**
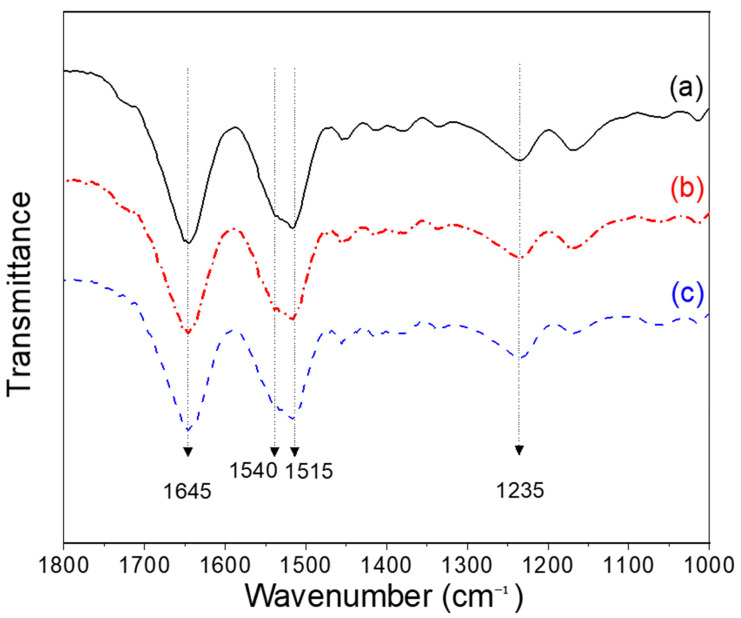
Effect of relative humidity on the Fourier transform infrared spectra of the electrospun regenerated silk web. Silk concentration was 17.5% at (**a**) 20%, (**b**) 40%, and (**c**) 60% relative humidity.

The molecular conformation of silk has been extensively examined because it affects the physical and chemical properties of silk. To investigate the effect of RH on the molecular conformation of the electrospun silk web, FTIR spectroscopy was used, and the result is shown in Figure 5.

In the amide, I band, an absorption peak appeared at 1645 cm^−1^. Two peaks, at 1515 and 1540 cm^−1^, are overlapped in the amide II band and a peak at 1235 cm^−1^ is shown in the amide III bands. The infrared (IR) absorption peaks at 1645, 1540, and 1235 cm^−1^ are attributed to the random coil conformation of the silk. On the other hand, the peak at 1515 cm^−1^ corresponds to β-sheet conformation [61,62,63,64,65,66]. This result showed that the electrospun silk web simultaneously has a random coil and β-sheet conformations. This result is consistent with that of silk web electrospun from silk formic acid dope solution [24,25,26]. As reported in a previous study, the partial β-sheet crystallization in the electrospun web from silk formic acid is due to (1) short flight time of polymer jet and (2) crystallization-inducing formic acid, i.e., formic acid induces crystallization of silk when the formic acid is eliminated from the silk [67]. However, the crystallization of silk is restricted because the silk molecules have insufficient time to be arranged. Finally, the silk is partially crystallized during electrospinning.

**Figure 6:**
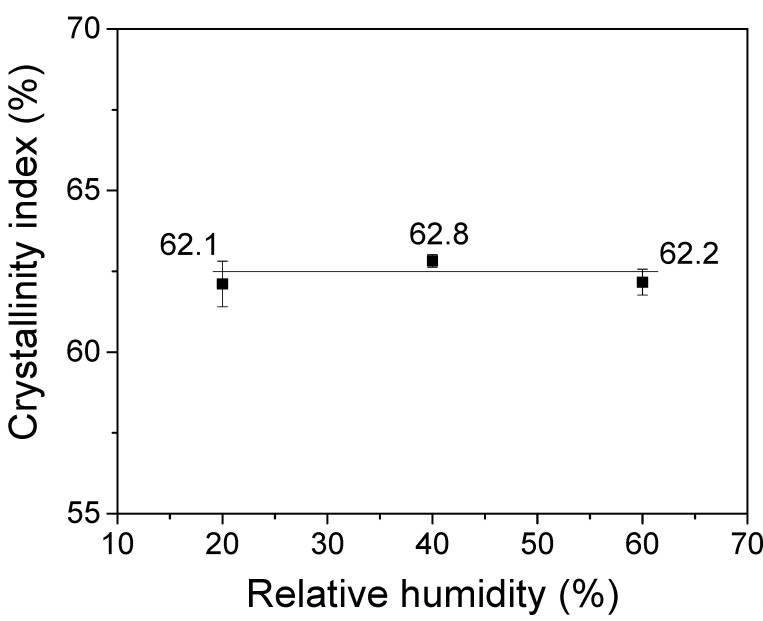
Effect of relative humidity on the crystallinity index of the electrospun regenerated silk web (*n* = 7). Silk concentration was 17.5%. The error bars represent the standard deviation.

No changes in the IR peaks of the electrospun silk web are observed due to RH during electrospinning. The crystallinity index from the amide III bands has been used to quantitatively examine the conformation and microstructure of silk [24,64]. Thus, although no significant change of IR appears in Figure 5, to quantitatively examine the effect of RH on the conformational change of electrospun silk web, the crystallinity index is calculated from FTIR spectra and the result is presented in Figure 6. The crystallinity index of the electrospun silk web does not change by RH, confirming the FTIR result of Figure 5. These results imply that the RH does not affect the crystallization of silk, which means there are no conformation changes in silk during electrospinning.

This might be due to the fact that the amount of water in the environment during electrospinning is not high enough to change the molecular conformation of electrospun silk, i.e., it was reported that the β-sheet conformation is induced by post-water or vapor treatment on the silk or regenerated silk nanofiber [68,69]. The water molecules act as a plasticizer, which promotes β-sheet formatting through helix–helix interaction. This interaction comes from the Gly-rich domain in the helix structures of silk, and it accelerates the β-sheet crystallization. In addition, it has previously been reported that the crystallinity index of silk is increased when the RH is over 90% [70]. Thus, an adequate amount of water (i.e., RH) in the electrospinning environment is essential to decide not only the structural properties of silk nanofiber but also the molecule conformation of silk. It seems that a maximum of 60% RH is insufficient to induce the additional crystallization of silk, except for the formic acid-induced β-sheet crystallization.

## 4. Conclusions

This study examined the effects of RH on the morphology of the electrospun silk web and the electrospinning rate of silk. In addition, the effect of RH on the molecular conformation of the electrospun silk web was examined. As RH was increased, the electrospinnability deteriorated, resulting in bead formation as well as decreases in the fiber diameter and maximum electrospinning rate. The molecular conformation of the electrospun silk web did not change with RH.

These results show that the morphology of electrospun silk fiber, including fiber diameter, can be diverse when controlling the RH. However, as the RH is increased, the production rate of the electrospun silk web can be decreased. When the RH is increased to 60%, problems including bead formation and low production rates of the electrospun silk web can occur. Thus, the RH of the electrospinning environment for silk should be carefully controlled to maintain the consistent quality and morphology of the electrospun silk web. In addition, the RH should be set at a low level (at least below 60%) to obtain a high production rate for the electrospun silk web.

## Figures and Tables

**Table 1:** Field emission-scanning electron microscopy photographs of electrospun silk fibers with various silk concentrations and relative humidities. The white magnification bars in the image represent 50 μm.

Relative Humidity (%)	Silk Concentration (%(*w*/*w*))			
	14	16	17.5	19
20	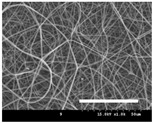	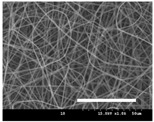	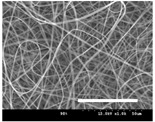	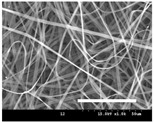
40	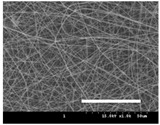	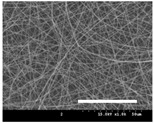	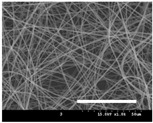	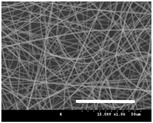
60	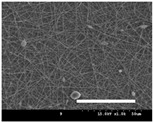	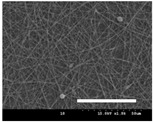	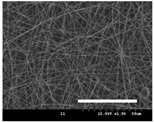	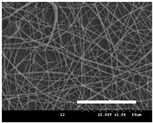

## Data Availability

The data presented in this study are available on request from the corresponding author.
